# Effects of a lipid-encapsulated zinc oxide supplement on growth performance and intestinal morphology and digestive enzyme activities in weanling pigs

**DOI:** 10.1186/2055-0391-56-29

**Published:** 2014-12-09

**Authors:** Insurk Jang, Chang Hoon Kwon, Duck Min Ha, Dae Yun Jung, Sun Young Kang, Man Jong Park, Jeong Hee Han, Byung-Chul Park, Chul Young Lee

**Affiliations:** The Regional Animal Industry Center, Gyeongnam National University of Science and Technology, Jinju, 660-758 Republic of Korea; College of Veterinary Medicine and Institute of Veterinary Science, Kangwon National University, Chuncheon, 200-701 Republic of Korea; R & D Institute, Sunjin Co., Ltd, 517-3 Doonchon-dong, Kangdong-gu, Seoul, 134-060 Republic of Korea

**Keywords:** Weanling pig, Zinc oxide, Dietary supplement, Growth, Diarrhea, Villus structure, Digestive enzyme

## Abstract

This study compared the effects of varying lipid content and dietary concentration of a lipid-encapsulated (LE) ZnO product to those of native ZnO and thereby to find insights into optimal lipid coating and dosage of the Zn supplement. A total of 192 21-d-old weanling pigs were allotted to 48 pens, after which each six pens received a ZnO-free basal diet supplemented with 125 ppm ZnO (100 ppm Zn; BASAL), 2,500 ppm Zn as native ZnO (HIGH), or 100 or 200 ppm Zn as LE ZnO (LE-100 or LE-250) containing 8%, 10%, or 12% lipid [LE-8%, LE-10%, or LE-12%, respectively; 2 × 3 factorial arrangement within the LE-ZnO diets (LE-ALL)] for 14 d. Forty pigs were killed at the end for histological and biochemical examinations. None of ADG, ADFI, gain:feed, and fecal consistency score differed between the LE-ALL and either of the BASAL and HIGH groups. Hepatic and serum Zn concentrations were greater (p <0.05) in the HIGH vs. LE-ALL group, but did not differ between LE-ALL and BASAL, between LE-100 and -250, or among LE-8%, -10%, and -12% groups. Villus height (VH), crypt depth (CD), and the VH:CD ratio in the duodenum, jejunum, and ileum did not differ between the LE-ALL and either of the BASAL and HIGH groups, except for a greater CD in the duodenum in the LE-ALL vs. HIGH group. Additionally, VH and CD in the duodenum and VH:CD in the jejunum were greater in the LE-250 vs. LE-100 group. Specific activities of sucrase, maltase, and leucine aminopeptidase in these intestinal regions and those of amylase and trypsin in the pancreas were not influenced by the lipid content or dietary concentration of LE ZnO and also did not differ between the LE-ALL and either of the BASAL and HIGH groups, except for a greater pancreatic amylase activity in the former vs. HIGH group. In conclusion, the present results indicate that the LE ZnO, regardless of its lipid percentage or supplementation level examined in this study, has no significant effect on growth performance, fecal consistency, or digestive enzyme activities of weanling pigs under the experimental conditions.

## Background

Zinc oxide (ZnO) is commonly supplemented to the pig starter diet to 2,000 to 3,000 ppm to prevent the post-weaning diarrhea as well as to enhance transiently retarded growth of post-weaning pigs [1–3]. However, supplementation of ZnO at this pharmacological level poses substantial concerns about environmental pollution, because dietary ZnO is mostly unabsorbed and therefore excreted into the environment via feces [4, 5]. This has led to limiting the in-feed ZnO concentration within 150 ppm by legislation in the European Union. Accordingly, some manufacturers have lately introduced new ZnO products which are more active or more efficiently delivered to the intestine than conventionally used ZnO, thereby suggesting the possibility of reducing the amount of ZnO added to the diet by substitution of the former for the latter.

A few types of non-covalent ZnO-carrier conjugates have been manufactured as a means of increasing the efficiency of ZnO delivery. In this regard, Hu et al. [6–8] have reported that 600 to 900 ppm supplementation of Zn as ZnO supported on zeolite or 500 ppm of Zn as a ZnO-smectite conjugate was as effective as 2,000 to 2,250 ppm of Zn as native ZnO in enhancing growth performance and digestive function of weanling pigs. HiZox (Animine, France) is also a ZnO product whose surface area is maximized to increase the bioavailability of the compound. As for its relative potency, Morales et al. [9] have reported that post-weaning pigs fed a diet supplemented with 110 ppm of Zn as HiZox exhibited a greater ADG, a greater G:F ratio, and a better health status than those fed the same diet supplemented with 2,500 ppm of Zn as ZnO; however, this needs to be confirmed.

The ZnO particle has also been coated with an enteric substance to increase the delivery of the mineral to the intestine [10–12]. Shield Zn (CTCBIO, Inc., Seoul) is a lipid-encapsulated ZnO product, which has been manufactured based on a rationale that the encapsulated ZnO particle reaches the intestine efficiently and is subsequently released upon digestion of the lipid capsule by lipase because unlike the inorganic ZnO, the mineral component of the product is not released as Zn^2+^ under acidic pH in the stomach owing to the outer enteric coating [12]. In this connection, we have found that dietary supplementation of 72 ppm of Zn as the LE ZnO (100 ppm) was as effective as 2,000 ppm of Zn as native ZnO (2,500 ppm) in alleviating reduced growth, diarrhea, and impaired integrity of intestinal mucosal structure in weanling pigs artificially infected with enterotoxigenic *Escherichia* coli (ETEC) K88 [11]. The present study was undertaken to investigate the effects of the LE ZnO relative to those of native ZnO as well as the effects of the lipid content and dietary concentration of the LE ZnO on growth and the measures of digestive function as an initial step to finding the optimal lipid coating and dosage of the Zn supplement in naïve weanling pigs.

## Methods

### Animals

The protocol for the present experiment was approved by the Institutional Animal Care and Use Committee (IACUC) of Gyeongnam National University of Science and Technology. The (Yorkshire × Landrace) × Duroc piglets were divided into the high-, medium- and low-body weight categories at weaning at 21 days of age. Ninety-six high- and 96 medium-body weight weanling pigs were randomly allotted to 48 pens by body weight to forty-eight 1-m^2^ nursery pens, with two females and two castrated males housed per pen equipped with a feeder and a nipple waterer. Each six pens received a ZnO-free basal nursery diet supplemented with 125 ppm of native ZnO (100 ppm ZnO; BASAL), 3,125 ppm of native ZnO (HIGH), 100 or 200 ppm Zn as LE ZnO (LE-100 or LE-250) containing 8%, 10%, or 12% lipid [LE-8%, LE-10%, or LE-12%, respectively; 2 × 3 factorial arrangement within the LE-ZnO diets (LE-ALL)] for 14 d (Table 1). The basal diet was formulated to contain a low percentage of crude protein to reduce the post-weaning diarrhea resulting from undigested proteins [10, 13]. The ambient temperature was maintained at 30°C up to d 7 and then lowered to 29°C. Fecal consistency was scored on d 1, 4, 7, and 14 according to an arbitrary 3-point integer scale as described by Heo et al. [13] and Zhao et al. [14]: 1, well-formed feces; 2, sloppy feces; 3, diarrhea.

**Table 1:** **Calculated chemical composition of the basal diet** ^**1)**^ **(as-fed basis)**

Item	Content
DE, MCal/kg	3.34
Crude protein (%)	16.50
Ether extract (%)	3.91
Lysine (%)	1.21
Zn (ppm)	100, 250, or 2,500

^1)^The composition of ingredients, which was grains-soy-whey-based, was reported previously [11]. Experimental diets were manufactured by supplementing the basal diet containing no Zn additive with 125 or 3,125 ppm of native ZnO or 139 to 355 ppm of Shield Zn® (CTCBIO, Seoul) encapsulated with 8%, 10%, or 12% lipid (w/w) to provide 100, 250, or 2,500 ppm of Zn.

### Collection of blood samples and intestinal tissues

A total of 40 pigs, which consisted of 16 pigs from the BASAL and HIGH groups (8 pigs each) and 24 pigs from the 6 LE groups (4 pigs each), were sacrificed as described previously [15]. Blood, pancreas, and intestinal tissues at the regions of the duodenum, jejunum, and ileum were collected also as described [11, 15].

### Determination of Zn

Five grams of hepatic tissue or 1 mL of serum was digested with 10 mL of 70% nitric acid at 150°C to complete solublization, filtered and diluted with 100 mL of distilled water. The Zn content in the digested and diluted solution was determined using an inductively coupled plasma atomic emission spectrophotometer (model 5300DV, Perkin-Elmer, Waltham, MA, USA).

### Histological and biochemical determinations

The intestinal tissue was fixed, embedded in paraffin, mounted on the glass slide, stained, and subjected to microscopic determination of the villus height and crypt depth as described previously [11, 15].

The intestinal mucosa and pancreatic tissue were homogenized and stored at −70°C until used. The protein content of the homogenate was determined using the bicinchronic acid protein assay kit (Pierce, Rockford, IL, USA); the specific activities of sucrase, maltase, leucine aminopeptidase, and trypsin were determined as described previously [15–17].

### Statistical analysis

All data were analyzed using the MIXED procedure of SAS (SAS Inst. Inc., Cary, NC, USA). The pen was the experimental unit in all variables, except for postmortem measurements in which the piglet was regarded as the experimental unit. The model included each dietary treatment as the main effect. In the analysis of repeated measurements, effects of the day and its interaction with the main effect were also included in the model. Accordingly, effects of the diet and the day including its interaction with the diet were tested using the experimental unit and experimental unit × day as error terms, respectively. In addition, effects of supplementation of LE ZnO vs. native ZnO as well as those of the lipid percentage and supplementation level of LE ZnO were analyzed by the contrast.

## Results

Growth performance including the average daily gain (ADG), average daily feed intake (ADFI), and gain:feed (G:F) ratio during the 14-d experimental period did not differ between the LE-ALL group and either of the BASAL and HIGH groups (Table 2). Moreover, within the LE-ALL group animals, the performance parameters were not different between the LE-10% group and either of the LE-8% and LE-12% groups or between the LE-100 and LE-250 groups.

**Table 2:** **Effects of supplementations of native ZnO and lipid-encapsulated ZnO (LE ZnO) on growth and fecal consistency of weanling pigs** ^1)^

Variable	Dietary supplementation (ppm as Zn)										Contrast ***P*** -value				
	Native ZnO		LE ZnO						SEM		LE ZnO vs.		Within LE ZnO		
	100	2,500	8% lipid ^2)^		10% lipid ^2)^		12% lipid ^2)^			***P*** -value	Native ZnO		10% lipid vs.		100 vs. 250
			100	250	100	250	100	250			100	2,500	8%	12%	
Growth performance															
Initial BW (kg)	6.69	6.98	6.76	6.67	6.71	6.65	6.74	6.73	0.15	0.85	0.92	0.05	0.78	0.67	0.63
Final BW (kg)	9.63	9.89	9.65	9.35	9.52	9.18	9.65	9.73	0.20	0.32	0.65	0.14	0.52	0.15	0.34
ADG (g)	210	207	207	192	201	180	208	214	11	0.49	0.50	0.60	0.49	0.11	0.34
ADFI (g)	355	367	355	329	344	327	348	369	13	0.24	0.49	0.13	0.60	0.09	0.51
G:F	0.591	0.569	0.582	0.586	0.581	0.538	0.603	0.579	0.025	0.76	0.63	0.74	0.34	0.21	0.30
Fecal consistency score^3)^															
Day 1	1.00	1.00	1.00	1.00	1.00	1.00	1.00	1.00							
Day 4	1.00	1.13	1.08	1.00	1.00	1.13	1.04	1.04	0.07^a^						
Day 7	1.04	1.00	1.04	1.00	1.04	1.13	1.13	1.17							
Day 14	1.17	1.00	1.17	1.17	1.25	1.35	1.17	1.08							
Overall^4)^	1.05	1.03	1.07	1.04	1.07	1.15	1.08	1.07	0.04	0.59	0.35	0.11	0.07	0.27	0.63

^1)^Data are means of 6 pens in each dietary group.

^2)^Denotes the percentage of lipid (w/w) encapsulating the ZnO particle.

^3)^Scored according to a 3-notch integer scale: 1, well formed; 2, sloppy; 3, diarrhea.

^4)^ *P*-values for the day and day × treatment were <0.01 and 0.71, respectively.

^a^Applies to all day × treatment combinations.

BW, body weight; ADG, average daily gain; ADFI, average daily feed intake; G:F, gain:feed.

The majority of the piglets exhibited well-formed feces throughout the experimental period, although the fecal consistency score (FCS) was greater (*P* <0.01) on d 14 than at any other time point (Table 2). However, the FCS did not differ between the LE-ALL group and either of the LBASAL and HIGH groups, between the LE-10% group and either of the LE-8% and -12% groups, or between the LE-100 and -250 groups.

The Zn concentration in the liver did not differ between the LE-ALL and BASAL groups (Table 3). However, hepatic ZnO concentration was 11-fold greater in the HIGH vs. LE-ALL group whereas within the LE-ALL group, it was not influenced by the lipid percentage or supplementation level of the LE ZnO. Serum Zn concentration, which did not differ between the LE-ALL and BASAL groups, was marginally greater in the HIGH group vs. LE-ALL (2.45 vs. 1.49 μg/mL; *P* <0.01). However, within the LE-ALL group, serum Zn concentration was not influenced by the lipid percentage or supplementation level of the ZnO product.

**Table 3:** **Effects of dietary supplementations of native ZnO and lipid-encapsulated ZnO (LE ZnO) on hepatic and circulating Zn concentrations, intestinal villus structure, and specific activities of digestive enzymes in weanling pigs**

Enzyme				Dietary supplementation (ppm as Zn)								Contrast ***P*** -value				
	Native ZnO ^1)^			LE ZnO							***P*** -value	LE ZnO vs.		Within LE ZnO		
	100	2,500	SEM	8% lipid ^2)^		10% lipid ^2)^		12% lipid ^2)^		SEM		Native ZnO		10% lipid vs.		100 vs.
				100	250	100	250	100	250			100	2,500	8%	12%	250
Zn concentration																
Liver (μg/g)	34.9	412.1	13.6	33.3	55.4	27.8	33.5	36.3	39.0	19.2	<0.01	0.87	<0.01	0.48	0.72	0.52
Serum (μg/ml)	1.78	2.45	0.14	1.53	1.63	1.52	1.50	1.14	1.62	0.22	<0.01	0.10	<0.01	0.75	0.57	0.31
Villus structure																
Duodenum																
VH (μm)	292	284	14	299	365	299	315	277	323	20	0.07	0.21	0.10	0.22	0.71	<0.01
CD(μm)	230	227	11	240	282	238	276	231	256	16	0.07	0.07	0.05	0.78	0.41	<0.01
VH:CD	1.27	1.26	0.04	1.25	1.29	1.26	1.15	1.20	1.26	0.05	0.56	0.37	0.66	0.21	0.60	0.98
Jejunum																
VH (μm)	280	252	16	254	315	240	266	275	263	21	0.31	0.54	0.38	0.15	0.46	0.17
CD(μm)	230	206	15	234	262	225	238	251	218	20	0.49	0.64	0.08	0.44	0.89	0.87
VH:CD	1.21	1.16	0.03	1.09	1.20	1.07	1.12	1.11	1.20	0.04	0.03	0.02	0.30	0.21	0.13	<0.01
Ileum																
VH (μm)	245	209	13	215	247	245	234	230	232	19	0.56	0.48	0.11	0.67	0.67	0.62
CD(μm)	228	186	13	205	221	238	224	238	180	19	0.15	0.73	0.05	0.33	0.24	0.24
VH:CD	1.10	1.21	0.07	1.30	1.16	1.03	1.05	1.09	1.31	0.11	0.36	0.54	0.50	0.08	0.15	0.71
Enzyme activity (μmol end product · mg protein^-1^ · min^-1^)																
Sucrase																
Duodenum	0.043	0.034	0.008	0.076	0.039	0.040	0.049	0.041	0.044	0.012	0.25	0.61	0.14	0.26	0.89	0.39
Jejunum	0.874	0.537	0.225	0.319	0.401	0.160	0.628	0.509	0.170	0.318	0.59	0.06	0.51	0.91	0.86	0.79
Ileum	0.098	0.117	0.032	0.105	0.099	0.088	0.107	0.094	0.119	0.046	1.00	0.91	0.68	0.93	0.86	0.74
Maltase																
Duodenum	5.18	4.41	1.10	8.15	4.56	5.98	7.71	5.34	7.80	1.56	0.36	0.28	0.10	0.76	0.86	0.88
Jejunum	15.87	12.70	3.48	8.35	12.95	10.28	14.10	13.88	5.38	4.92	0.76	0.22	0.64	0.76	0.61	0.99
Ileum	4.20	5.39	1.49	5.11	4.02	2.91	6.27	4.13	3.95	2.11	0.96	0.91	0.57	0.99	0.80	0.69
LAP																
Duodenum	2.00	2.30	0.36	2.19	1.91	1.99	2.72	1.90	3.20	0.50	0.53	0.45	0.96	0.55	0.70	0.17
Jejunum	3.15	2.73	0.27	2.37	2.53	3.17	2.51	3.25	2.20	0.38	0.30	0.14	0.86	0.31	0.77	0.11
Ileum	1.76	1.93	0.29	2.04	1.71	1.68	1.94	1.61	1.81	0.41	0.99	0.90	0.69	0.88	0.81	0.89
Amylase (PAN)	241.2	142.1	23.0	262.1	185.1	224.3	240.7	237.6	231.4	31.4	0.06	0.67	<0.01	0.78	0.95	0.39
Trypsin (PAN)	0.156	0.107	0.022	0.149	0.127	0.150	0.184	0.160	0.197	0.032	0.43	0.86	0.06	0.37	0.71	0.53

^1)^Data are means of 8 animals in each group.

^2)^Denotes the percentage of lipid (w/w) encapsulating the ZnO particle. Data are means of 4 animals in each group.

VH, villus height; CD, crypt depth; LAP, leucine aminopeptidase; PAN, pancreas.

The villus height (VH) and crypt depth (CD) in the LE-ALL group were not different from those in the BASAL and HIGH groups in the duodenum (Table 3). Within the LE-ALL group, these morphology variables did not change due to a change in the lipid percentage of the LE ZnO, but increased when the supplementation level was increased from 100 ppm Zn to 250 ppm (*P* <0.01). However, the VH:CD ratio in the duodenum did not differ between the LE-ALL group and either of the BASAL and HIGH groups, between the LE-10% group and either of the LE-8% and -12% groups, or between the LE-100 and -250 groups. In the jejunum, the VH and CD did not differ between the LE-ALL group and either of the BASAL and HIGH groups and also were not influenced by the lipid percentage or supplementation level of the LE ZnO. However, the VH:CD ratio was greater (*P* <0.05) in the BASAL group (1.21) than in the LE-ALL group (1.13), within which it was greater (*P* <0.01) in the LE-250 vs. LE-100 group (1.17 vs. 1.09). In the ileum, the CD was greater in the LE-ALL vs. HIGH group (218 vs. 186 μm); otherwise, the villus morphology variables did not differ between the LE-ALL group and either of the BASAL and HIGH groups, between the LE-100 and -250 groups, or among the LE-8%, -10%, and -12% groups.

Specific activities of sucrase, maltase, and leucine aminopeptidase in the mucosa were not affected by any treatment factor examined in the present study in any intestinal segment (Table 3). Specific activities of amylase and trypsin in the pancreas also did not change due to any treatment factor, except for a greater amylase activity in the LE-ALL vs. HIGH group.

## Discussion

The present results indicated that neither supplementation level of native ZnO or LE ZnO nor lipid percentage of the latter has any significant effect on ADG, ADFI, G:F, or FCS. However, this does not necessarily mean that neither pharmacological supplementation of native ZnO nor the basal-level supplementation of LE ZnO has any significant effect on these performance parameters, because even the known growth-enhancing effect of the former [2, 3, 18, 19] was not apparent under the present experimental conditions. As a matter of fact, we have observed a growth-enhancing effect of the LE ZnO supplemented at a basal level (100 ppm as Zn concentration; unpublished results). The lack of growth-enhancing effects of the LE ZnO and HIGH treatments in the present study is therefore reflective of the known fact that growth enhancers including ZnO are less effective under experimental settings than under production conditions [20, 21]. In this context, the present results were also reminiscent of the lack of effects of the in-feed antibiotics, the known growth enhancers in swine [20, 21], on growth performance of post-weaning pigs placed in small experimental pens in our previous study [15]. As such, more studies are warranted to determine the optimal usage of the LE ZnO as a growth-enhancing dietary supplement for weanling pigs under production conditions.

The several-fold and marginally greater Zn concentrations in the HIGH vs. BASAL group in the liver and serum, respectively, were consistent with published results [22–24]. Moreover, neither circulating Zn concentration was different between the LE ZnO and BASAL groups as in a report of Kim et al. [10], nor hepatic Zn concentration was influenced by the lipid coating. These results imply that the supplementation level of ZnO is well reflected into the Zn concentration in the liver where many heavy metals are stored and that the absorption rate of Zn at the intestine is not influenced by lipid coating of the ZnO particle.

The lack of effect of the LE ZnO or HIGH treatment on the intestinal villus structure was quite different from the increase in the VH and/or VH:CD ratio as well as the decrease in CD in response to either treatment in weanling pigs artificially infected with ETEC K88 in our previous study [11]. Furthermore, the positive effect of pharmacological ZnO on the integrity of the villus structure often observed in weanling pigs [25–27] also was not apparent in the present study. These results are thus interpreted to suggest that the beneficial effect of either Zn supplement on the integrity of the villus may be apparent only when the villus structure of the piglet is substantially damaged by any causative like the microbial infection. In this context, the increased VH in the duodenum and increased VH:CD in the jejunum in response to the LE-250 treatment vs. LE-100 observed in the present study, albeit intriguing, needs to be rigorously confirmed to make any firm conclusion as to the dose effect of the LE ZnO on the villus structure in naïve weanling pigs.

Results on the digestive enzyme activities in the intestinal mucosa and pancreatic tissue also indicated that these are not affected by the supplementation level of the LE ZnO or native ZnO or by the lipid percentage of the former, although the pancreatic amylase activity was greater in the LE-ALL vs. HIGH group. Similarly, in the study of Hedemann et al. [28], effects of the high-ZnO (2,500 ppm as Zn) diet on pancreatic and intestinal enzyme activities in weanling pigs were inconclusive or equivocal.

## Conclusions

The present results indicated that neither the high-ZnO supplementation nor the physiolgical supplementation of the LE ZnO containing 8 to 12% lipid to 100 or 250 ppm Zn has any significant effect on growth performance, fecal consistency, villus morphology, or digestive enzyme activities of the piglets under the experimental conditions. More studies are necessary, however, to determine the effects of the LE ZnO relative to those of native ZnO on these measures in weanling pigs under production conditions.
